# Supplementary material for: Adapting new norms: A mixed-method study exploring mental well-being challenges in dental technology education
Source: PLoS One. 2025 Mar 25;20(3):e0320602. doi: 10.1371/journal.pone.0320602 (PMC11936265; doi:10.1371/journal.pone.0320602)
Supplement: Attachment — Submitted filename: Response to Reviewers.pdf [file pone.0320602.s001.pdf]

Response to Reviewers

The authors would like to thank the editor and reviewers for considering the manuscript and for their valuable comments. The amendments are made in the revised manuscript.

No.	Reviewer comment	correction
Reviewer 1		
1.	Please attach semi structured interview guide.	Thank you for your valuable feedback. We appreciate your suggestion regarding the inclusion of the semi-structured interview guide. We have now added the interview guide in **Table 1** for your reference.<br><br>Page 9:<br><br>*"Table 1. Interview topic guide questions"*
2.	Please elaborate the validity of the guide.	Thank you for your thoughtful feedback regarding the validity of the interview guide. We have elaborated on this aspect by including the following sentences in the manuscript.<br><br>Page 8:<br><br>"*Due to the limited number of BDT students in Malaysia, a pre-test was conducted with five Bachelor of Dental Surgery (BDS) students, whose similar academic environments and learning experiences made them a suitable proxy. A subsequent pilot test with 10 BDS students further refined the guide's face validity, allowing for adjustments to improve clarity and usability. While the target population was BDT students, the overlap in educational challenges between BDS and BDT students during the pandemic justified this approach, ensuring the guide was well-prepared for the final interviews.*"<br><br>We hope this addition clarifies the steps taken to ensure the validity of the interview guide. Thank you for your valuable input and consideration.
3.	Why phenomenological approach was employed?	Phenomenology was employed in this study because it focuses on investigating and comprehending individuals' lived

		experiences. Given the aim to explore students' emotional and personal experiences during the COVID-19 pandemic, this approach allowed for a deeper understanding of how they perceived and navigated academic challenges. Phenomenology is well-suited to capture the richness and complexity of these experiences, providing valuable insights into the unique ways students adapted to and overcame obstacles in their learning journey.
4.	Please align conclusions with the findings of the study.	We have modified the conclusion as suggested by both reviewers.
5.	Please review the themes and subthemes and consider presenting in a tabular form if feasible	Thank you for your valuable feedback regarding the presentation of themes and sub-themes. We have carefully reviewed and revised the themes and sub-themes. This adjustment enhances the clarity and readability of the qualitative findings, while preserving the original meaning and integrity of each theme and sub-theme. As Figure 1 effectively conveys the necessary information, we believe that adding a table would result in redundancy. Nonetheless, we appreciate your insightful suggestion, which has contributed to improving the overall presentation of the manuscript.
6.	Please mention the framework utilised for qualitative analysis	Thank you for your insightful comment. We would like to clarify that the qualitative analysis in this study was guided by Braun and Clarke's Thematic Analysis Framework. This approach was selected for its flexibility and ability to identify, analyze, and report patterns directly from the data, allowing for a rich, detailed exploration of students' experiences without being restricted by a rigid theoretical model.<br><br>We have added two sentences to the methodology section.<br><br>Page 9:<br><br>"*The qualitative data were then analyzed using Braun and Clarke's Thematic Analysis Framework, which allows for a flexible yet systematic exploration of*

		patterns and themes that emerge from the data. This approach is well-suited for identifying recurring themes without being bound to a rigid theoretical structure."
Reviewer 2		
1.	Thank you for the great effort in this article. It seems you have spent an enormous amount of time conducting this study. The main advantage of such study is its unique population.	Thank you for highlighting the uniqueness of the study population. We believe this focus on dental technology students will contribute valuable insights to the field of allied health profession.
2.	Mention the number of participants and response rate if available.	We have addressed this by stating the number of participants in the results section.<br><br>Page 10:<br><br>*"All 10 eligible students (100% response rate) ...."*
3.	Clarify the aim of the qualitative part.	We have addressed your comment by adding the aim of the qualitative study to the last paragraph of the introduction section.<br><br>Page 5:<br><br>"*Hence, the present qualitative study aimed to explore the lived experiences of dental technology students during the post-COVID-19 pandemic transition*."
4.	In the conclusion, report findings related to the levels of depression, anxiety, and stress.	We have refined the conclusion by adding the general findings.<br><br>Page 21:<br><br>"*While most students experienced normal to mild levels of stress and depression, a notable proportion reported moderate to severe anxiety*."
5.	Rephrase "content validation" as "content was validated".	We have rephrased the sentence.<br><br>Page 7:<br><br>"*The content of the questionnaire was validated by two faculty experts…*"
6.	**Introduction:**	Thank you for your insightful feedback. We have added a citation from a previous

	Provide a citation for the claim "may find themselves at the intersection of healthcare and technology."	qualitative study we conducted among dental technologists to substantiate this statement.<br><br>We acknowledge the limited literature available on dental technologists' perceptions, and this has been highlighted within the manuscript. We hope this study contributes to increasing awareness among readers and healthcare professionals about the importance of addressing the welfare of allied dental professionals.
	Clarify whether "dental technology students" refers only to Malaysia or if it applies globally.	We would like to clarify that this study specifically focuses on dental technology students in Malaysia. However, as mentioned in the introduction, we believe that the findings can serve as a benchmark for similar programs globally, providing valuable comparative perspectives.
	Add citations for "Challenges in the hybrid learning method" and "Change in study-life balance."	The phrases "*Challenges in the hybrid learning method*" and "*Change in study-life balance*" are actually sub-themes derived from our qualitative findings, as detailed in the Results section. However, we appreciate your attention to this point, and we would like to highlight that these sub-themes are further elaborated in the Discussion section, where appropriate references and relevant literature are cited to support and contextualise our findings.
	The introduction has some redundancy and could be more concise. Highlight studies that have investigated the psychological health of dental technologists. For example, consider including studies like this one to establish previous research in this area.	We greatly appreciate your suggestion to highlight studies on the psychological health of dental technologist students. However, after conducting an extensive literature search across PubMed, Scopus, and Google Scholar using various keywords [("dental technologist" OR "dental technician") AND ("mental health" OR "psychological health" OR "well-being" OR "stress" OR "anxiety" OR "depression")], we found very limited studies focusing specifically on depression, anxiety or stress among dental technologist students or dental technicians. This gap in the literature highlights the novelty and importance of our study.

		In addition, Malaysia is the only country in Southeast Asia offering a Bachelor's degree in Dental Technology, further highlighting the uniqueness of this population and the challenges in drawing comparisons or citing similar studies. We believe this study contributes to addressing this gap and provides valuable insights for future research in this underrepresented area.
7.	**Methods:**<br><br>The "COVID-19 Status During the Study" section should be shortened. Include only the study period in brief. This section reads as though it was derived from a thesis, so be more concise and adapt it to fit a journal article.	Thank you for your valuable feedback on the "COVID-19 Status During the Study" section. We agree with your point that a more concise version aligns better with the format of a journal article. However, we would like to note that previous reviewers recommended elaborating on this aspect to provide a clearer understanding of the context and circumstances during the study period.<br><br>Nevertheless, we have taken your suggestion into account and have summarised this section.<br><br>Page 6:<br><br>"*On 8th March 2022, Malaysia's Prime Minister announced the country's transition to the endemic (post-pandemic) phase and the reopening of borders on 1st April 2022 ….. The study was conducted approximately 8 months after the transition to the endemic phase.*"
	"At the time of the study conducted, there were only 10 BDT students for the cohort 2019-2023 throughout the entire country" – Do you mean there are only 10 students in this specialty across all cohorts? Please clarify.	At the time the study was conducted, there were only 10 Bachelor of Dental Technology (BDT) students for the 2019-2023 cohort across the entire country. The BDT program at AIMST University is the only undergraduate dental technology program in Malaysia, with an annual intake of fewer than 10 students. This highlights the rarity and significance of this group.<br><br>Furthermore, across Southeast Asia, this is the only bachelor-level dental technology program available, further emphasising the exclusivity of this program and its unique position within the region. We believe that investigating the mental health and

		academic challenges faced by this distinct and underrepresented group will provide valuable insights that could inform educational strategies and policies, not just in Malaysia but potentially for similar programs globally.
	Explain what modifications were made to the questionnaire. The DASS-21 is widely used without modification. Clarify why changes were necessary.	Thank you for your insightful comment regarding the modifications made to the DASS-21 questionnaire. We appreciate your attention to this detail.<br><br>In the manuscript, we mentioned that the DASS-21 was adapted and modified to suit the context of dental technology students. As shown in **Table 3**, general terms of each questionnaire item were adjusted to explicitly refer to "*dental technology students*" to enhance clarity and applicability within this unique cohort.<br><br>We believe these contextual adjustments preserve the integrity of the original scale while ensuring the questionnaire reflects the experiences of our target population.
	Instead of "faculty experts," specify their specialties and qualifications (e.g., Master's, PhD).	We have revised the manuscript to provide greater clarity regarding the qualifications and specialties of the experts involved in the validation process.
	For a sample size of 10 students, using factor loadings and Cronbach's alpha might not be meaningful due to the small sample size. Consider reporting only descriptive statistics.	Regarding the appropriateness of factor loadings and Cronbach’s alpha, we completely understand the concern about the limited statistical power associated with a sample of 10 students. However, during an earlier phase of the review process, previous reviewers recommended including factor analysis and Cronbach’s alpha to demonstrate the internal consistency and validity of our adapted questionnaire, even with the small sample. In addition, we consulted our university’s biostatistician, who advised us to proceed with these analyses to provide an overview of the psychometric properties of the instrument. We acknowledge the limitations associated with small-sample factor analysis and have therefore interpreted the results with caution. We appreciate your understanding

		and are open to further guidance if necessary.
	Participant recruitment for qualitative interviews should be made more concise. Mention the sampling method (e.g., purposeful sampling).	We have revised the participant recruitment section, by adding phrase indicating 'one-on-one' interviews and the sampling method.<br><br>Page 8:<br><br>"*A convenience sampling method was used and only those who were readily available and willing to participate were recruited*."
	When referring to "WWT" and "GSSL," define them the first time you mention them (provide abbreviations for the investigators).	To ensure investigator anonymity, we have revised the manuscript to refer to "GSSL" and "WWT" as the first and second investigators, respectively.
8.	**Results:**<br><br>Summarise the quantitative results briefly, and if reporting Cronbach's alpha, do so without exaggerating the findings (e.g., "indicate robust reliability across the questionnaire items" is too strong for such a small sample).	We appreciate your suggestion regarding the presentation of the quantitative results. In response, we have revised the sentence.<br><br>Page 10:<br><br>"*Cronbach's alpha values for depression (0.81), anxiety (0.84), and stress (0.90) indicated acceptable reliability*."
	Remove Table 1 and present the data in the text, as there are only a few variables.	We have removed Table 1 as suggested and have presented the demographic data within the text.<br><br>Page 10:<br><br>"*Most (60%) of the students were aged between 21 and 22, while 20% were aged 23 to 24, and another 20% were aged 25 to 26.*"
	Avoid redundancy in reporting the results. For example, rather than repeating text from Table 2, simply state, "The prevalence of depression, anxiety, and stress among BDT students is shown in Table 2."	We appreciate your suggestion regarding reducing redundancy in reporting the results. In response, we have summarized the paragraph.<br><br>Page 10:<br><br>"*Most students experienced normal levels of depression (60%), anxiety (30%), and stress (70%). Moderate depression was reported by 30%, while anxiety levels varied, with*

		30% experiencing severe to extremely severe symptoms. No cases of extremely severe depression and stress were noted."<br><br>This revision reduces word count while maintaining clarity and conciseness.
	The extensive changes made to the DASS-21 may affect whether the study is measuring depression, anxiety, and stress as intended, or whether it is measuring aspects more related to the profession. I recommend focusing on measuring depression, anxiety, and stress, and leaving the contextual analysis to the qualitative part.	We would like to clarify that the modifications made to the DASS-21 were minimal and did not alter the core content or structure of the instrument. The changes primarily involved adapting the terminology to reflect the context of dental technology students, ensuring relevance without deviating from the original meaning. To maintain the integrity of the scale, the modified version underwent content validation by two experts with experience in dental education and psychometric evaluation. This step ensured that the questionnaire remains a reliable measure of depression, anxiety, and stress as originally intended. We appreciate your attention to detail and trust this clarification addresses your concern.
	Indicate whether the data were normally distributed; otherwise, use median and IQR	Thank you for your valuable feedback regarding the data distribution. We have conducted a normality test (Shapiro-Wilk) for each domain of the DASS-21 scores. The results indicate that the data were normally distributed. Given that the p-values for all domains exceed 0.05, we have proceeded with reporting the mean and standard deviation for the quantitative results.<br><br>Page 10:<br><br>"*Based on the Shapiro-Wilk normality test, the data were normally distributed across all domains: Depression (W=0.943, p=0.482), Anxiety (W=0.967, p=0.763), and Stress (W=0.921, p=0.305)*."
	Avoid redundancy throughout the article, such as repeating "Only six students agreed to take part in the interview sessions," which has been mentioned earlier.	Upon reviewing the manuscript, we have decided to remove the sentence "Only six students agreed to take part in the interview sessions" as suggested to avoid redundancy.

		We appreciate your careful attention to detail.
	The qualitative data analysis should be more in-depth than the quantitative part, as it is critical to the study's findings.	Thank you for your valuable feedback regarding the qualitative data analysis. We understand the importance of in-depth qualitative insights for this study. However, due to the unique nature of the dental technology program in Malaysia, there are currently only 10 students enrolled in the entire country. Out of this small cohort, six students voluntarily agreed to participate in the interview sessions.<br><br>While we acknowledge that the qualitative sample size may be limited, we believe this study sheds light on an underrepresented group within the allied health professions. By publishing this article, we hope to raise awareness and encourage further research and support for dental technology students. Unfortunately, the absence of additional Bachelor of Dental Technology students nationwide limits our ability to recruit more participants at this time.<br><br>We sincerely appreciate your understanding and consideration of these constraints.
9.	**Discussion:**<br><br>Avoid over-exaggerating the validity and consistency of the questionnaire. You can discuss these aspects if you conducted a pilot with a larger sample (e.g., 12 participants) before using the questionnaire in your main study.	In response to your suggestion, we have removed the sentences:<br><br>*"The high internal consistency observed in the reliability assessment of the questionnaire items, as indicated by Cronbach's alpha values exceeding the 0.7 threshold, lends credibility to the findings. In addition, the acceptable values in confirmatory factor analysis further support the validity of the measurement tool, affirming the appropriateness of the chosen domains for assessing mental well-being among dental technology students."*<br><br>Nonetheless, we added this into the discussion.<br><br>Page 19:

		"*While the internal consistency of the questionnaire items yielded acceptable Cronbach's alpha values (all exceeding the 0.7 threshold), these results should be interpreted with caution due to the small sample size. The confirmatory factor analysis also demonstrated acceptable values; however, the limited participant pool restricts the generalizability of the results*."<br><br>We appreciate your recommendation to avoid over-exaggerating the validity of the questionnaire, given the small sample size.
	In the second paragraph, clarify whether the Malaysian and Italian studies you referenced involved dental students or dental technology students specifically.	Thank you for your thoughtful feedback. We appreciate the opportunity to clarify this point.<br><br>The Italian study referenced in the discussion involved students from a range of disciplines, including Medicine and Surgery, Psychology, Biology, Pharmacy, Economics, Engineering, and Social and Political Sciences, reflecting the broader impact of the pandemic on higher education students.<br><br>As noted earlier in the manuscript, the Bachelor of Dental Technology program is exceptionally rare, and dental technology students are underrepresented in existing literature. This scarcity makes direct comparisons with published studies challenging. However, to address this concern, we have highlighted the uniqueness of this cohort and the importance of raising awareness about this underrepresented group.<br><br>We believe that the insights gained from this study contribute valuable knowledge to the field and emphasize the need for more research focused on dental technology students.
	Reduce repetition in the manuscript. For example, the implications and recommendations for dental education are repeated in different paragraphs.	Thank you for your thoughtful feedback regarding the repetition of implications and recommendations in the manuscript. In response, we have removed one of the

	Combine these into one cohesive section to avoid redundancy	paragraphs and the key points from this section have been merged into the final paragraph of the discussion to provide a more cohesive presentation of the study's implications.<br><br>We believe this revision enhances the overall readability and avoids unnecessary repetition. We are grateful for your valuable insights and hope that the updated manuscript reflects these improvements.
